# [^68^Ga]Ga-PSMA PET/MRI, histological PSMA expression and preliminary experience with [^177^Lu]Lu-PSMA therapy in relapsing high-grade glioma

**DOI:** 10.3389/fonc.2022.980058

**Published:** 2022-09-02

**Authors:** Peter Truckenmueller, Josefine Graef, Michael Scheel, Peter Vajkoczy, David Capper, David Kaul, Christian Furth, Holger Amthauer, Winfried Brenner, Julia Sophie Onken

**Affiliations:** ^1^ Department of Neurosurgery, Berlin Institute of Health, Charité - Universitätsmedizin Berlin, Corporate Member of Freie Universität Berlin, Humboldt-Universität zu Berlin, Berlin, Germany; ^2^ Department of Nucelar Medicine, Berlin Institute of Health, Charité - Universitätsmedizin Berlin, Corporate Member of Freie Universität Berlin, Humboldt-Universität zu Berlin, Berlin, Germany; ^3^ Department of Neuroradiology, Berlin Institute of Health, Charité - Universitätsmedizin Berlin, Corporate Member of Freie Universität Berlin, Humboldt-Universität zu Berlin, Berlin, Germany; ^4^ Department of Neuropathology, Charité - Universitätsmedizin Berlin, Freie Universität Berlin, Humboldt-Universität zu Berlin, Berlin, Germany; ^5^ German Cancer Consortium (DKTK), Partner Site Berlin, German Cancer Research Center (DKFZ), Heidelberg, Germany; ^6^ Department of Radiation Oncology, Berlin Institute of Health, Charité - Universitätsmedizin Berlin, Freie Universität Berlin, Humboldt-Universität zu Berlin, Berlin, Germany; ^7^ Berlin Institute of Health at Charité – Universitätsmedizin Berlin, Berlin, Germany

**Keywords:** IHC PSMA expression, relapsing malignant glioma, [177Lu]Lu-PSMA therapy, [68Ga]Ga-PSMA PET/MRI, individualized treatment

## Abstract

**Purpose:**

High-grade gliomas (HGG) are still associated with a dismal prognosis. Prostate specific membrane antigen (PSMA) is discussed as a theranostic target for PSMA-directed radioligand therapy ([^177^Lu]Lu-PSMA RLT). Here, we report on the correlation of [^68^Ga]Ga-PSMA uptake with histological PSMA expression and on our preliminary experience with [^177^Lu]Lu-PSMA RLT in relapsing HGG.

**Methods:**

Patients with relapsing HGG underwent [^68^Ga]Ga-PSMA PET/MRI to evaluate eligibility for an individualized treatment approach with [^177^Lu]Lu-PSMA. Standard uptake values (SUV) for tumor and liver and respective tumor-to-background ratios (compared to the liver) (TBR) on [^68^Ga]Ga-PSMA PET/MRI were assessed. Eligibility criteria for [^177^Lu]Lu-PSMA therapy were exhaustion of all standard treatment options available and TBR_max_>1.0. In 11 samples, immunohistochemical PSMA expression was determined, quantified using the H-score and correlated with uptake on [^68^Ga]Ga-PSMA PET/MRI.

**Results:**

We included 20 patients with a median age of 53 years (IQR 42-57). The median SUV on [^68^Ga]Ga-PSMA PET/MRI was 4.5 (3.7-6.2) for SUV_max_ and 1.4 (1.1-1.7) for SUV_mean_. The respective TBR was maximum 0.6 (0.4-0.8) and mean 0.3 (0.2-0.4). High TBR_max_ correlated with increased endothelial PSMA expression [H-score of 65 (62.5-77.5)]. Three patients (15%) presented a TBR_max_>1.0 and qualified for [^177^Lu]Lu-PSMA RLT. No treatment related toxicity was observed.

**Conclusion:**

Only a minority of patients with relapsing HGG qualified for [^177^Lu]Lu-PSMA RLT. Our data demonstrates that PSMA expression in the neo-vasculature corresponds to PSMA uptake on [^68^Ga]Ga-PSMA PET/MRI and might be used as a screening tool for patient selection. Future prospective studies need to focus the debate on TBR_max_ thresholds as inclusion criteria for PSMA RLT.

## Introduction

High-grade gliomas (HGG) are highly invasive and rapidly growing primary brain tumors and account for 81% of all malignant CNS tumors (1). Glioblastoma is the most common and aggressive form and still associated with a sustained morbidity and dismal prognosis, with a median survival time of less than two years (2, 3). However, despite tremendous efforts to identify and develop innovative therapeutic approaches, surgery followed by radiotherapy with concomitant and adjuvant *Temozolomide* (TMZ) still remains the standard of care (2–4). In relapsing HGG, no substantial therapeutic advances could be achieved either. Salvage options are limited to further resection, reirradiation and systemic therapies like *Lomustine* that have failed to show durable effects (5). Within recent years, there is an increasing interest in exploring theranostic targets in HGG which may serve not only as diagnostics but also as targets of radioligand therapy (RLT). Multiple studies demonstrated that PSMA is expressed in the tumor-associated neo-vasculature of HGG and might interact with different pathways promoting angiogenesis (6–10). In prostate cancer, there is a strong correlation between PSMA expression and PSMA tracer uptake on PET imaging. For HGG, data from smaller case series (1-30 patients) support the use of [^68^Ga]Ga-HBED-CC-PSMA or [^68^Ga]Ga PSMA-11 PET for noninvasive assessment of PSMA expression for diagnosis and disease monitoring (11–16). However, no analysis of the correlation between immunohistological PSMA expression and tracer uptake on PET/CT or PET/MRI has been published yet for HGG.

The role of salvage PSMA RLT has been evaluated in multiple retrospective observational studies in metastatic prostate cancer. Previously published trials on PSMA RLT, including a phase III clinical trial, have confirmed promising response rates and low toxicity in patients with PSMA-positive metastatic castration-resistant prostate cancer, especially when added to the standard care (17–20). In the context of HGG, only two published case reports exist to date describing the first experience with PSMA RLT in relapsing glioblastoma (21, 22). In the current study, we analyzed [^68^Ga]Ga-PSMA PET/MRI results of a cohort 20 HGG patients and correlated PET/MRI findings with immunohistochemical PSMA expression. Further, we report our preliminary experience with [^177^Lu]Lu-PSMA salvage therapy in three cases with relapsing HGG.

## Materials and methods

### Patient cohort and clinical data assessment

In this mono-institutional, retrospective cohort study, patients with relapsing HGG underwent [^68^Ga]Ga-PSMA PET/MRI to evaluate eligibility for an individualized treatment approach with [^177^Lu]Lu-PSMA from 05/2021 to 02/2022. Data on patients´ medical history and previous therapies were collected and immunohistological expression of PSMA was assessed in a subset of patients with sufficient tumor tissue available for further analysis. The following clinical and pathological information was collected: WHO diagnosis including isocitrate dehydrogenase (IDH) status and O(6)-methylguanine-DNA methyltransferase (MGMT) promoter status, age at diagnosis, gender, number and kinds of previous therapies and number of recurrences.

### [^68^Ga]Ga-PSMA PET/MRI and [^177^Lu]Lu-PSMA therapy

[^68^Ga]Ga-PSMA PET/MRI scans were performed using a Siemens Biograph mMR (VE11P; Siemens Healthcare GmbH, Erlangen, Germany). PET data were acquired after injection of median 177 (170.5 – 185) Megabecquerel (MBq) [^68^Ga]Ga-PSMA and a tracer uptake time of 68 (60.0 – 87.5) minutes. The PET scan was acquired in each scan with a single bed position covering first the whole skull and afterwards the liver (3D list mode acquisition). The ultrashort echo-time sequence (UTE) was used for attenuation and scatter correction in MRI of the head. For the MRI of the liver, we used MR based attenuation correction (MRAC) including a CAIPIRINHA-accelerated T1-weighted DIXON 3D-VIBE sequence. Both sequences were provided by Siemens Healthcare (Siemens Healthcare GmbH, Erlangen, Germany). We used the ordered-subset expectation maximization (OSEM; 3 iterations, 21 subsets; head: voxel matrix 344 x 344 x 127; voxel spacing 1.0 x 1.0 x 2.0 mm; liver: voxel matrix 172 x 172 x 127; voxel spacing 4.2 x 4.2 x 2.0 mm) for reconstruction of PET raw data. MRI sequences of the head included, among individually adjusted sequences, T2 weighted turbo spin echo (TSE), diffusion weighted imaging (DWI), apparent diffusion coefficient (ADC) sequence and post contrast 3D magnetization prepared for rapid acquisition with gradient echo (MPRAGE). ADC values of the tumors were determined using a region of interest (ROI) of five millimeters. Diffusion restrictions in the DWI within the tumors were analyzed and dichotomized into whether a diffusion restriction was present or not present. Standard uptake value (SUV) was measured in the tumor lesion and the liver. The analysis of maximum and mean SUV (SUV_max_/SUV_mean_) was performed with ROVER (v2.1.12, ABX GmbH, Radeberg, Germany) using the attenuation corrected (AC) PET data. Background ROI of the liver were drawn manually. To determine the tumor ROI, we used an automatic threshold-based ROI definition by setting the SUV_mean_ of the superior sagittal sinus as lower cut-off without upper limitation. In case of spatial spread tumor manifestations, multilocal tumor ROIs were defined separately and were combined afterwards. Visual control of physiological tracer uptake within the tumor ROI was performed and incorrect background uptakes were excluded manually when necessary. The maximum and mean tumor-to-background ratios (TBR) compared to the liver were evaluated for all patients as reported by Kunikowska et al. (21).

According to the standard treatment protocol in our clinic for patients with prostate cancer, the median treatment activity in patients who qualified for the [^177^Lu]Lu-PSMA therapy was 6.03 (5.74 – 6.10) gigabecquerel (GBq). For each treatment cycle patients received a dosimetry protocol (data not presented) including planar whole-body scintigrams 2 hours (h), 24 h and 48 h post injection (p.i.) and a single photon emission computed tomography/low-dose CT (SPECT/CT) of the head, thorax, abdomen and pelvis was performed 48 h p.i.

### Immunohistochemical procedures

Immunohistochemical staining was performed on a Benchmark XT autostainer (Ventana Medical Systems, Tuscon, AZ, USA) with standard antigen retrieval methods (CC1 buffer, pH8.0, Ventana Medical Systems, Tuscon, AZ, USA) using 4-μm-thick FFPE tissue sections. The following primary antibody was used: mouse monoclonal anti-PSMA (1:50, NCL-L-PSMA, clone 1D6, Leica Biosystems). The iVIEW DAB Detection Kit (Ventana Medical Systems, Tuscon, AZ, USA) was used according to the manufacturer’s instructions. Sections were counterstained with hematoxylin, dehydrated in graded alcohol and xylene, mounted and coverslipped. IHC stained sections were evaluated by an experienced neuropathologist (DC). The extent of PSMA immunoreactivity of cytoplasm was evaluated using the semiquantitative H-score (23). In order to calculate the H-score, both the intensity of the PSMA staining graded as 0, 1, 2 or 3 corresponding to non-, weak, intermediate or strong staining and the percentage of positive cells with the respective staining grade were assessed. Calculation of the H score resulted in scores between 0 and 300, with 300 representing a strong staining grade of 100% of the cells (24, 25). The H-score was evaluated separately for tumor cells, endothelial cells, periendothelial cells (cells belonging to the vascular structures besides endothelial cells) and other cells within the tumor area (mostly inflammatory cells and reactive cells).

### Ethical statement

This study was conducted according to the ethical principles of medical research involving human subjects according to the Declaration of Helsinki. The clinical data were assessed and anonymized for patients’ confidentiality. Ethical approval (EA4/060/22) was granted by the institutional ethics board of the Charité Ethics Committee.

### Statistical analysis

Data were expressed as median and interquartile ranges (IQR). Statistical significance was measured using unpaired, two-tailed Student’s *t*-Test, assuming both populations having the same standard deviation. Significance level was set at alpha = 0.05 (95% confidence intervals). A multivariable linear regression model was used to analyze the relationship between the PSMA uptake using the SUV_max_ of the tumor, the immunohistological PSMA expression in the endothelium using the H-score and the ADC value of the [^68^Ga]Ga-PSMA PET/MRI. A multivariable logistic regression analysis was used to predict a potential relationship between the presence of a diffusion restriction in the DWI (dichotomized into diffusion restriction present or not present), the PSMA uptake using the SUV_max_ of the tumor and the immunohistological PSMA expression in the endothelium using the H-score. Quantitative and qualitative statistical analysis was performed using SPSS (Version 25, IBM Corporation, USA) and GraphPad Prism (Version 9, GraphPad Software Inc, USA). The graphs were created using GraphPad Prism (Version 9, GraphPad Software Inc, USA).

## Results

### Patient characteristics

We included 20 patients who underwent a [^68^Ga]Ga-PSMA PET/MRI with a median age of 53 (42-57) years and a female to male ratio of 1:3. Seventeen patients (85%) were diagnosed with an IDH wildtype glioblastoma multiforme (GBM), WHO grade 4, one patient showed an IDH mutant Astrocytoma, two patients suffered from a WHO grade 4 and two patients from an IDH mutant Astrocytoma WHO grade 3. Twelve patients (60%) showed hypermethylation of the MGMT promoter (**Table 1**). Of the 20 patients, two patients (10%) underwent one, seven patients (35%) underwent two, two patients (10%) underwent three, five patients (25%) underwent four and four patients (20%) underwent five previous therapies before the [^68^Ga]Ga-PSMA PET/MRI was conducted. The median number of recurrences prior to the [^68^Ga]Ga-PSMA PET/MRI was 3 (2-3) with 2 (1-2) reoperations (**Table 1**).

**Table 1:** Demographics and clinical characteristics.

Patients	Overall	20 (100%)
Age at diagnosis	Median (IQR)	53 (42-57)
Sex	Female Male	5 (25%) 15 (75%)
Diagnosis/WHO grade	Glioblastoma, IDH wildtype/4 Astrocytoma, IDH mutant/4 Astrocytoma, IDH mutant/3	17 (85%) 1 (5%) 2 (10%)
MGMT	Methylated Unmethlyated Not specified	2 (10%) 12 (60%) 6 (30%)
First Line Surgery	Resection Biopsy only	19 (95%) 1 (5%)
Number of reoperations prior to [^68^Ga]Ga-PSMA PET/MRI	0 1 2 3	1 (5%) 9 (45%) 7 (35%) 3 (15%)
Number of recurrences prior to [^68^Ga]Ga-PSMA PET/MRI	1 2 3 4 > 4	3 (15%) 4 (20%) 8 (40%) 4 (20%) 1 (5%)

IDH, Isocitrate dehydrogenase; MGMT, O(6)-methylguanine-DNA methyltransferase.

### Semiquantitative data from [^68^Ga]Ga-PSMA PET/MRI

The maximum standard uptake value (SUV_max_) of the tumor was 4.48 (3.73 – 6.20) and the median of SUV_mean_ was 1.43 (1.13 – 1.67). The median uptake values in the background liver were SUV_max_ 8.05 (IQR 6.53 – 10.40) and SUV_mean_ 4.25 (3.53 – 6.50). For the treatment decision the respective ratios of tumor-to-liver were evaluated as follows, TBR_max_ 0.57 (0.36 – 0.80) and median TBR_mean_ 0.32 (0.23 – 0.42). Three patients presented a maximum TBR > 1.00 (1.35; 1.06; 1.03). In those patients, the SUV_max_ was 8.65, 7.97 and 6.39 in the tumor and 6.40, 7.50 and 6.20 in the liver, respectively. The median SUV_max_ was 8.0 (6.4-8.3) in the tumor and 6.4 (6.2-7.0) in the liver (**Table 2**).

**Table 2:** SUV and TBR in [^68^Ga]Ga-PSMA PET/MRI.

	Overall	[^177^Lu]Lu-therapy		p-value
		Not eligible	Eligible	
Patients	20 (100%)	17 (85%)	3 (15%)	
Tumor size in cm^3^				
Contrast enhancement FLAIR	20.9 (10.9-44.5) 122.4 (69.8-154.5)	16.4 (9.5-32.9) 120.5 (74.2-150.6)	49.1 (30.9-54.6) 144.7 (100.6-188.7)	0.19 0.62
Tumor SUV_mean_ Tumor SUV_max_	1.4 (1.1-1.7) 4.5 (3.7-6.2)	1.4 (1.1-1.6) 4.3 (3.4-4.9)	1.7 (1.5-1.9) 8.0 (6.4-8.3)	0.11 0.00
Liver SUV_mean_ Liver SUV_max_	4.3 (3.5-6.5) 8.1 (6.5-10.4)	4.5 (3.7-7.0) 8.5 (7.0-10.8)	3.6 (3.2-3.9) 6.4 (6.2-7.0)	0.15 0.20
Tumor-to-liver ratio				
TBR_mean_ TBR_max_	0.3 (0.2-0.4) 0.6 (0.4-0.8)	0.3 (0.2-0.7) 0.5 (0.4-0.7)	0.5 (0.4-0.6) 1.1 (1.0-1.2)	0.01 0.00
Previous therapies	3 (2-4)	3 (2-4)	3 (2.5-3.5)	0.94

FLAIR, Fluid-attenuated inversion recovery; SUV, standard uptake values; TBR, tumor-to-background ratio.

The overall median value of the tumor in the ADC sequence was 1309 (925-1644). The median tumor ADC value of the patients not eligible for the [^177^Lu]Lu-PSMA RLT was 1358 (1008-1633) and 922 (856-1433) for the three patients who underwent the [^177^Lu]Lu-PSMA RLT (*p* = 0.72). Five of the 17 patients (29.4%) who did not qualify for the [^177^Lu]Lu-PSMA RLT and 1/3 patients (33.3%) who qualified for the [^177^Lu]Lu-PSMA RLT showed a diffusion restriction of the tumor in the DWI (*p* = 0.89). A multivariable linear regression analysis found no relationship of the ADC value of the [^68^Ga]Ga-PSMA PET/MRI with the SUV_max_ of the tumor (*p* = 0.50; *t* = 0.70; *F* = 0.50) or with the H-score of the immunohistochemical endothelial PSMA expression (*p* = 0.67; *t* = 0.45; *F* = 0.20), with an R^2^ of 0.06. Further, logistic regression did not show a relationship between the presence of a diffusion restriction within the tumor in the DWI (dichotomized into diffusion restriction present or not present) and the PSMA uptake using the SUV_max_ of the tumor (odd´s ratio 0.93; 95% CI 0.45-1.84) or the H-score of the immunohistochemical endothelial PSMA expression (odd´s ratio 1.01; 95% CI 0.96-1.06).

### Only a minor proportion of patients diagnosed with HGG qualifies for [^177^Lu]Lu-PSMA RLT

A total of three patients with a maximum TBR of 1.35, 1.06 and 1.03 were subjected to [^177^Lu]Lu-PSMA RLT. One patient was diagnosed with an IDH wildtype glioblastoma, WHO grade 4, one with an IDH mutant astrocytoma, WHO grade 4 and one IDH mutant astrocytoma, WHO grade 3. The RLT was the third-, fourth- and fifth-line therapy after new recurrence was revealed in the follow-up MRI. Each patient received two treatment cycles with a median activity dose of 6.03 GBq (5.74 – 6.10) according to the standard treatment protocol in our clinic for patients with prostate cancer. The time intervals between the two cycles were 10, 11 and 9 weeks. The follow-up period for the three patients undergoing the RLT were 10, 11 and 15 weeks after the first cycle. All three patients received end-of-life palliative care right after the second cycle of the RLT due to further clinical and neurological deterioration. No treatment related toxicity was noted. One patient presented with chronic thrombocytopenia associated with a pre-existing myelodysplastic syndrome (CTCAE v5.0°II). Due to the short follow up period (maximum 15 weeks), no efficacy data can be provided yet.

### IHC PSMA staining and PSMA uptake on [^68^Ga]Ga-PSMA PET/MRI

**Figure 1:**
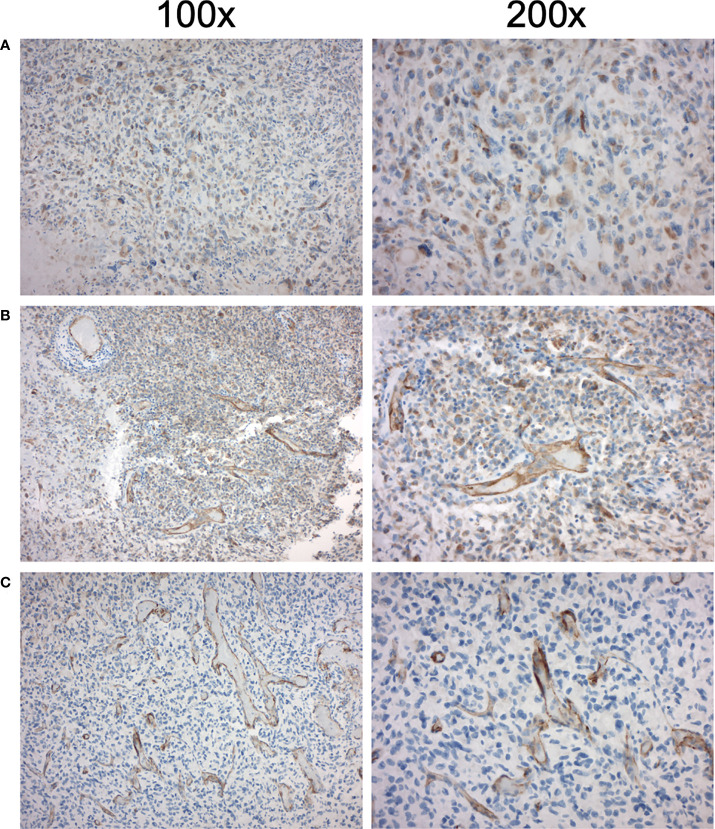
Immunohistochemical PSMA expression in high-grade glioma of three patients eligible for [^177^Lu]Lu-PSMA therapy. Left column 100x magnification, right column 200x magnification. **(A)** First patient with increased endothelial PSMA expression with H-score 60 and TBR_max_ of 1.03 **(B)** Second patient with increased endothelial PSMA expression with H-score 65 and TBR_max_ of 1.06 **(C)** Third patient with increased endothelial PSMA expression with H-score 90 and TBR_max_ of 1.35.

**Figure 2:**
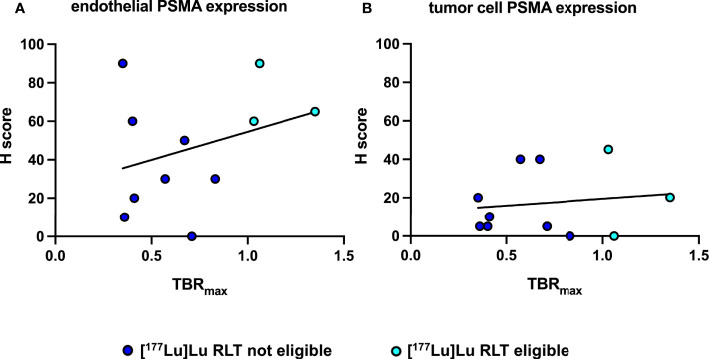
Correlation and simple linear regression analysis of immunohistochemical PSMA expression and [^68^Ga]Ga-PSMA uptake of patients eligible and not eligible for [^177^Lu]Lu RLT. **(A)** Between endothelial PSMA expression and [^68^Ga]Ga-PSMA uptake, *f* = 1.03 and *p* = 0.34 **(B)** Between tumor cell PSMA expression and [^68^Ga]Ga-PSMA uptake, *f* = 0.17 and *p* = 0.69.

**Figure 3:**
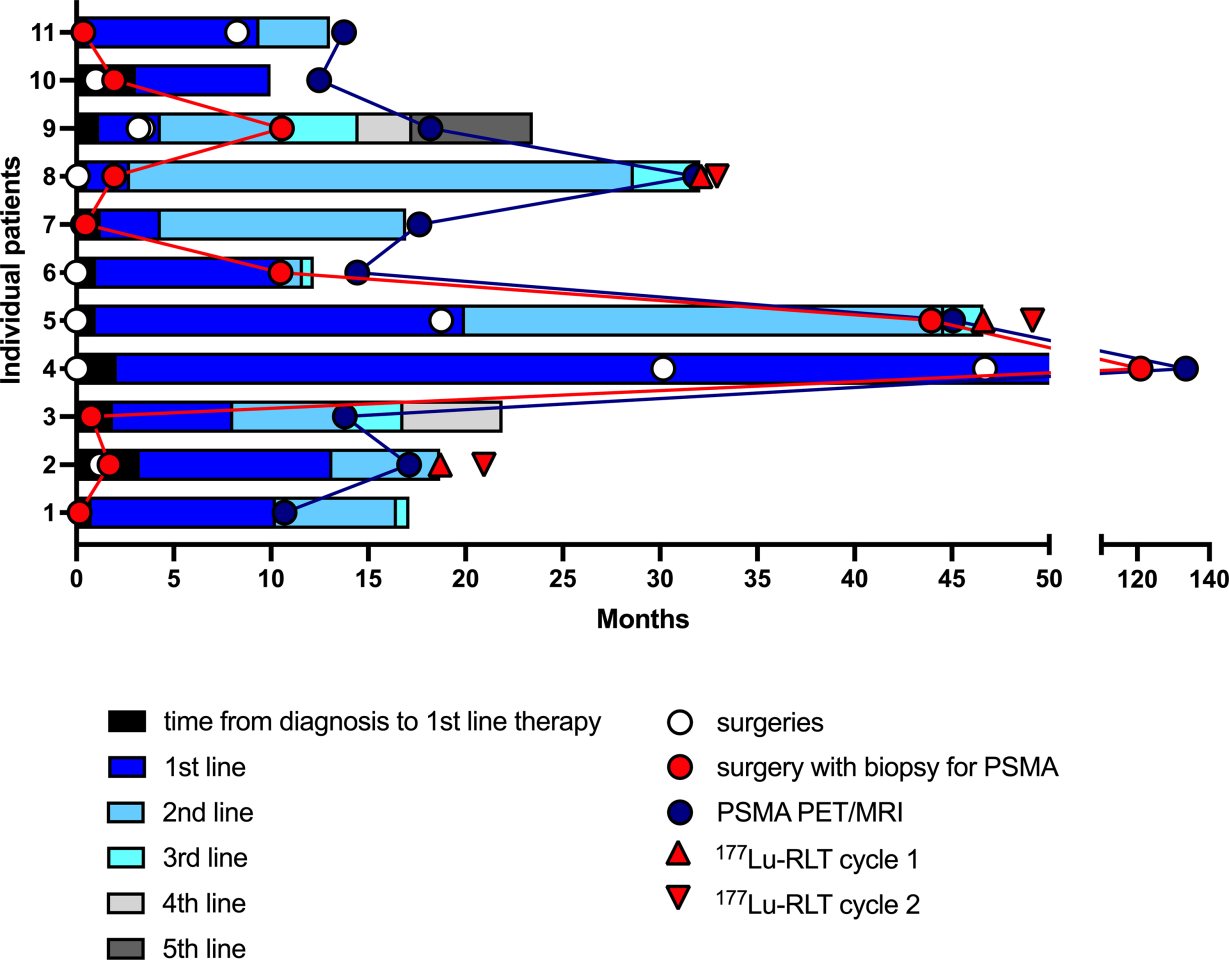
Swimmer plot showing the time interval between the biopsy with immunohistological PSMA staining and the PSMA PET/MRI in the context of previous therapies and surgeries of the eleven patients with available histological PSMA staining. Each section of the stacked columns reflects the period from beginning a therapy until initiation of the next line therapy. PSMA, prostate specific membrane antigen; ^177^Lu-RLT, [^177^Lu]Lu-PSMA RLT.

IHC PSMA expression was assessed in 11 patients (55%) (**Table 3**). Here, PSMA staining was predominantly found in endothelial cells of the tumor-associated neo-vasculature but not in periendothelial cells and less in tumor and other cells (**Figure 1**). Accordingly, the overall median PSMA expression in terms of the H-score was highest in endothelial cells with 50 (25-62.5) compared to 10 (5-30) in tumor cells, 10 (10-20) in non-tumor cells and 0 in periendothelial cells. In the three patients eligible for [^177^Lu]Lu-PSMA therapy, PSMA staining exhibited a higher H-score of 65 (62.5-77.5) in endothelial cells compared to 30 (17.5-52.5) in the patients who were not eligible for [^177^Lu]Lu-PSMA therapy (*p* = 0.08) and a linear regression analysis showed a trend for higher endothelial but not tumor cell PSMA expression in patients with increased [^68^Ga]Ga-PSMA uptake (**Figure 2**). The higher H-scores seemed to be associated with increased PSMA uptake in the [^68^Ga]Ga-PSMA PET/MRI. However, in patients with available immunohistological PSMA evaluation, the median time from tissue biopsy with PSMA staining to [^68^Ga]Ga-PSMA PET/MRI was 12.6 (9.1-14.4) months. A swimmer plot showing the time interval between the biopsy and the [^68^Ga]Ga-PSMA PET/MRI in the context of previous therapies is presented in **Figure 3**.

**Table 3:** Immunohistochemical PSMA expression.

	Overall	[^177^Lu]Lu- therapy		
		Not eligible	Eligible	p-value
Patients, n (%)	20 (100%)	17 (85%)	3 (15%)	
PSMA IHC available, n (%)	11 (55%)	8 (47.1%)	3 (100%)	
H-score				
Endothelial cells Periendothelial cells Tumor cells Other non-tumor cells	50 (25-62.5) 0 10 (5-30) 10 (10-20)	30 (17.5-52.5) 0 7.5 (5-25) 10 (8.8-20)	65 (62.5-77.5) 0 20 (10-32.5) 20 (15-30)	0.08 >0.99 0.73 0.35

IHC, immunohistochemistry; PSMA, prostate specific membrane antigen.

### Illustrative case

**Figure 4:**
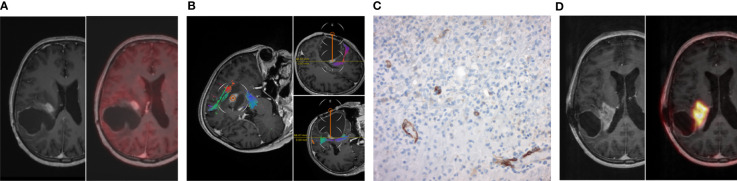
Illustrative case. **(A)** Preoperative MRI post contrast enhancement with standard [^18^F]Fluoro-ethyl-tyrosine (FET) PET/MRI right before open biopsy. **(B)** In-line view of the open biopsy trajectory. **(C)** Corresponding PSMA staining of the open biopsy with predominant endothelial PSMA expression. **(D)** MRI post contrast enhancement with [Ga^68^]Ga-PSMA PET/MRI only two months after the biopsy with maximum tumor-to-liver ratio of 1.35.

In one case of a 59 year old patient with an IDH mutant and MGMT methylated GBM, tumor biopsy was performed 2 months prior to [^68^Ga]Ga-PSMA PET/MRI. Biopsy trajectory is shown in **Figure 4** including the corresponding PSMA staining and [^68^Ga]Ga-PSMA PET/MRI. Immunohistological staining exhibited a strong and primarily endothelial PSMA expression with an H-score of 90. The high IHC PSMA expression correlated well with the increased PSMA uptake on [^68^Ga]Ga-PSMA PET/MRI, showing a SUV_max_ of 7.6 and a TBR_max_ of 1.4. Due to the increased TBR_max_, this patient was subjected to [^177^Lu]Lu-PSMA RLT as an individual treatment approach and completed two treatment cycles.

## Discussion

The three main novel findings of the current study are: i) that only a minor proportion of HGG patients qualifies for [^177^Lu]Lu-PSMA RLT based on TBR_max_ of tumor and liver >1. ii) Our histological studies exhibited variable PSMA expression but suggested a correlation of high PSMA expression in the tumor neo-vasculature in patients with increased PSMA uptake of the tumor on [^68^Ga]Ga-PSMA PET/MRI. iii) Future studies need to focus the debate on TBR_max_ thresholds as inclusion criteria for PSMA RLT and include patients at an earlier stage of their disease.

HGG are still associated with a dismal prognosis and especially in patients with recurrent gliomas, therapeutic options remain limited (2–4). In the recent years, immunohistological investigations demonstrated an increased histological PSMA expression in gliomas and further studies focused on radiolabeled PSMA ligands in the assessment of HGG (16). These findings do not only demonstrate the diagnostic value of radiolabeled PSMA ligands, but also suggest that radiolabeled PSMA PET/MRI might be used to identify patients with an increased PSMA uptake and who might benefit from a PSMA RLT. In line with the current literature, SUV and TBR in our study are significantly lower in HGG compared to prostate cancer (26). This may be due to the overall low PSMA expression in tumor cells, predominant expression of PSMA in the tumor neo-vasculature and the strong heterogeneity of these tumors. As a result, only a minority of recurrent HGG patients qualifies for [^177^Lu]Lu-PSMA RLT when the TBR of tumor and liver are used as selection criteria (21, 27). Such high TBRs allow for optimal tumor irradiation without exceeding recommended thresholds for other organs with physiological PSMA expression such as liver, salivary and lacrimal glands, kidneys, spleen, intestines and urinary bladder (28, 29). Previously published trials on PSMA RLT, including a phase III clinical trial, have confirmed promising response rates and low toxicity in patients with PSMA-positive metastatic castration-resistant prostate cancer (17–20). In our study, only three patients qualified for the PSMA RLT and the post-treatment period was too short to reliably assess the incidence of toxic effects. However, the follow-up period after the first treatment cycle was 10-15 weeks and no relevant adverse effects could be noted. Our study demonstrates the feasibility of the [^177^Lu]Lu-PSMA RLT in HGG patients and prospective studies are warranted to evaluate if [^177^Lu]Lu-PSMA RLT can be a therapeutic option in HGG. However, these studies need to discuss lower TBR_max_ thresholds as inclusion criteria and should include patients at an earlier stage of their disease, in order to evaluate the treatment response and toxicity.

Regarding the toxicity, evolving technologies like targeted alpha therapy, will be of great interest in the future (30). Due to the preliminary endothelial PSMA expression in the neo-vasculature of the tumor, radiation might affect tumor cells only in the immediate proximity to the vessels and the beta emitting radionuclide ^177^Lu with a pathlength of 2 mm might cause relevant irradiation of the peritumoral brain parenchyma. Alpha emitters have a very short pathlength and might deliver systemic radiation selectively to cancer cells while minimizing systemic toxic effects (31).

Multiple studies have proven the predominantly vascular expression of PSMA in HGG (32, 33). With this study, we are the first to correlate PSMA expression patterns in HGG with PSMA uptake on [^68^Ga]Ga-PSMA PET/MRI. By demonstrating stronger endothelial PSMA expression in patients with higher TBR on [^68^Ga]Ga-PSMA PET/MRI, immunohistochemical PSMA staining might allow for a targeted selection of patients with a higher likelihood of increased uptake in the PSMA PET/MRI and who might benefit from a [^177^Lu]Lu-PSMA RLT. However, the results must be interpreted with caution, since the time span between tumor biopsy and PET imaging in our study diverged widely and we report on a small number of patients with a heterogenous disease. Previous studies did not only demonstrate interindividual variability but also temporal changes of PSMA expression between initial diagnosis and recurrence (6, 34). Further, the heterogeneity of the tumor could also be a bias for a false-high or false-low PSMA staining depending on the localization of the biopsy. In order to establish a valid correlation between PSMA expression and PSMA uptake on [^68^Ga]Ga-PSMA PET/MRI, diagnostics should be obtained in a timely manner before the tumor resection. To correct for sampling bias, biopsy location should be correlated topographically with a potential hot spot in the [^68^Ga]Ga-PSMA PET/MRI in order to draw reliable conclusions.

### Limitations

The retrospective design of our study is subject to several well-known limitations. First of all, we included all subsequent patients with relapsing or progressive HGG who underwent a radiolabeled [^68^Ga]Ga-PSMA PET/MRI. Therefore, we have a heterogeneous patient population regarding previous therapies and number of recurrences at the time of [^68^Ga]Ga-PSMA PET/MRI. Second, the time span between tumor biopsy with immunohistological PSMA staining and the PET/MRI imaging in our study diverged, making it difficult to establish a direct correlation of IHC PSMA positivity and PSMA uptake on [^68^Ga]Ga-PSMA PET/MRI. Lastly, only patients with very limited life expectancy due to progressive HGG received [^177^Lu]Lu-PSMA RLT, which did not allow a clinical follow up beyond the 2^nd^ cycle.

## Conclusion

[^177^Lu]Lu-PSMA therapy is a promising theranostic approach in the treatment of HGG, a tumor entity still associated with an unacceptably poor prognosis. The fact that only a minority of patients undergoing [^68^Ga]Ga-PSMA PET/MRI were eligible for this therapy highlights the importance of reliable screening tools with clear thresholds. Prospective trials are needed to evaluate effectiveness and toxicity.

## Data availability statement

The raw data supporting the conclusions of this article will be made available by the authors, without undue reservation.

## Ethics statement

The studies involving human participants were reviewed and approved by Charité Ethics Committee. Written informed consent for participation was not required for this study in accordance with the national legislation and the institutional requirements.

## Author contributions

All authors contributed to the study conception and design. Material preparation, data collection and analysis were performed by PT, JSO, JG, MS and DC. The first draft of the manuscript was written by PT, JSO and JG and all authors commented on previous versions of the manuscript. All authors agree to be accountable for the content of the work.

## Funding

The authors declare that no funds, grants, or other support were received during the preparation of this manuscript.

## Conflict of interest

The authors declare that the research was conducted in the absence of any commercial or financial relationships that could be construed as a potential conflict of interest.

## Publisher’s note

All claims expressed in this article are solely those of the authors and do not necessarily represent those of their affiliated organizations, or those of the publisher, the editors and the reviewers. Any product that may be evaluated in this article, or claim that may be made by its manufacturer, is not guaranteed or endorsed by the publisher.
